# Cardiac and skeletal muscle predictors of impaired cardiorespiratory fitness post-anthracycline chemotherapy for breast cancer

**DOI:** 10.1038/s41598-021-93241-5

**Published:** 2021-07-07

**Authors:** Amy A. Kirkham, Mark J. Haykowsky, Rhys I. Beaudry, Justin G. Grenier, John R. Mackey, Edith Pituskin, D. Ian Paterson, Richard B. Thompson

**Affiliations:** 1 Faculty of Kinesiology and Physical Education, University of Toronto, Toronto, Canada; 2 Faculty of Medicine and Dentistry, University of Alberta, Edmonton, Canada; 3 Faculty of Nursing, University of Alberta, Edmonton, Canada

**Keywords:** Breast cancer, Chemotherapy

## Abstract

This study aimed to characterize peak exercise cardiac function and thigh muscle fatty infiltration and their relationships with VO_2_peak among anthracycline-treated breast cancer survivors (BCS). BCS who received anthracycline chemotherapy ~ 1 year earlier (n = 16) and matched controls (matched-CON, n = 16) were enrolled. Resting and peak exercise cardiac function, myocardial T_1_ mapping (marker of fibrosis), and thigh muscle fat infiltration were assessed by magnetic resonance imaging, and VO_2_peak by cycle test. Compared to matched-CON, BCS had lower peak SV (64 ± 9 vs 57 ± 10 mL/m^2^, p = 0.038), GLS (− 30.4 ± 2.2 vs − 28.0 ± 2.5%, p = 0.008), and arteriovenous oxygen difference (16.4 ± 3.6 vs 15.2 ± 3.9 mL/100 mL, p = 0.054). Mediation analysis showed: (1) greater myocardial T_1_ time (fibrosis) is inversely related to cardiac output and end-systolic volume exercise reserve; (2) greater thigh muscle fatty infiltration is inversely related to arteriovenous oxygen difference; both of which negatively influence VO_2_peak. Peak SV (R^2^ = 65%) and thigh muscle fat fraction (R^2^ = 68%) were similarly strong independent predictors of VO_2_peak in BCS and matched-CON combined. Post-anthracyclines, myocardial fibrosis is associated with impaired cardiac reserve, and thigh muscle fatty infiltration is associated with impaired oxygen extraction, which both contribute to VO_2_peak.

## Introduction

Elevated cardiovascular risk and impaired cardiorespiratory fitness (VO_2_peak) are now considered hallmarks of early stage breast cancer survivorship^1–4^. Understanding the mechanisms of impaired VO_2_peak is required to develop effective prevention and treatment strategies to reduce cardiovascular risk. Reduced VO_2_peak may be due to a lower peak exercise cardiac output (CO, product of stroke volume, SV, and heart rate, HR) and/or a lower arteriovenous oxygen difference (avO_2_diff), which indicates lower oxygen extraction by the exercising muscles. The direct cardiotoxic effects from treatment for breast cancer, especially anthracycline-based chemotherapy, is assumed to be the primary factor underlying these impairments^4^. Two prior studies, using indirect estimates or submaximal measures of SV during exercise, suggest that anthracycline chemotherapy has a detrimental effect on exercise SV in BCS^2,5^. One study has also reported that calculated avO_2_diff with submaximal exercise is reduced by anthracycline chemotherapy^5^.

Magnetic resonance imaging (MRI) is the gold standard for quantification of resting and exercise cardiac function and enables acquisition of complementary central and peripheral metrics with implications for VO_2_peak, including global longitudinal strain (GLS), and tissue characterization of the myocardium and the exercising thigh muscles (such as T_1_ mapping metric of fibrosis and fatty infiltration, respectively). The longitudinal shortening of the left ventricle (LV) is a primary determinant of SV^6^, and while resting GLS is a sensitive marker of anthracycline-related myocardial injury^7^, it is unknown whether exercise GLS is related to VO_2_peak. Diffuse myocardial fibrosis as measured by T_1_ mapping is another reported manifestation of anthracycline-related myocardial injury^8^. We previously reported strong associations between elevated myocardial T_1_ values with reduced VO_2_peak in anthracycline-treated BCS^9,10^, but etiology of the associations is not understood. Recently, we and others have also reported associations between non-cardiac parameters, specifically, fatty infiltration of the thigh muscles, with VO_2_peak following anthracycline therapy^10,11^.

We performed a comprehensive MRI study with an overarching goal of characterizing peak exercise cardiac function, including GLS, myocardial T_1_ time, and thigh muscle fatty infiltration (measured by fat fraction) and their relationships with VO_2_peak among anthracycline-treated BCS. The first objective was to compare peak exercise cardiac function among anthracycline-treated BCS, age- and body mass index (BMI)-matched non-cancer controls (matched-CON), and normative values for young, normal BMI, healthy controls (young-CON). We hypothesized that peak exercise cardiac function would be impaired in BCS compared to both matched-CON and young-CON. The second objective was to determine the relative contributions of central and peripheral factors to VO_2_peak with the hypothesis that both would be important predictors of VO_2_peak in BCS. The final objective was to determine whether exercise cardiac function and avO_2_diff mediate the relationship between cardiac and skeletal muscle tissue characteristics and VO_2_peak.

## Methods

### Design

This was a cross-sectional comparison of the three groups outlined below.

### Participants

We have previously reported skeletal muscle composition, bioenergetics, and oxygenation and gated-segmented cardiac MRI assessment of resting LV function and structure of the participants in this study^9,10^. In this manuscript we report exercise and reserve cardiac function and uncover underlying mechanisms for reduced VO_2_peak.

#### BCS

We mailed letters to a random selection of 75 women from 240 who received anthracycline-based chemotherapy for breast cancer at the Cross Cancer Institute (Edmonton, Canada) in the previous calendar year (2017). Exclusion criteria were: diagnosed cardiovascular disease, diabetes, lung disease, MRI contraindications. Twenty-two (29%) women responded and six were excluded (MRI contraindication, diabetes, not interested, n = 2 each); 16 participants were enrolled. One participant received doxorubicin (250 mg/m^2^) and the rest received epirubicin (median dose: 300 mg/m^2^) 12.8 ± 4.7 months prior; none received trastuzumab.

#### Matched controls

We used word-of-mouth and posters to recruit a woman without a history of cancer to match each BCS within two years of age and whenever possible, to within 3.0 kg/m^2^ of BMI. The same exclusion criteria were applied.

#### Normative data

Normative female peak exercise cardiac function is not well quantified. Therefore, we recruited a convenience sample of 12 young (22–32 years), healthy (BMI = 19–24.9 kg/m^2^, no cardiovascular risk factors) women to perform the resting and peak exercise cardiac MRI only to disentangle the influence of cardiovascular risk factors beyond cardiotoxic treatment. Cardiovascular risk factors were defined as sedentary behaviour, hypertension, diabetes, dyslipidemia, overweight/obese, and smoking status.

### MRI examination

MRI examinations were performed on a 3 T Siemens Prisma system with a 36-element chest/back array for signal reception (Siemens Healthcare, Erlangen, Germany). We used the SAturation recovery single SHot Acquisition (SASHA) pulse sequence to acquire native (non-contrast) T_1_ mapping for a single mid-basal short-axis slice at end-expiration^12^. SASHA acquisition parameters were: 0.96 ms echo time, 2.18 ms repetition time, 70° flip angle, 8 mm slice thickness, field of view 360 × 294 mm, acquisition matrix 208 × 128, 1415 Hz/pixel, rate 2 parallel imaging (GRAPPA) with acquisition during diastasis, and 10 saturation recovery images with a fixed recovery time of 630 ms. Real-time, free-breathing resting and exercise cine acquisitions of the left ventricle included six contiguous slices each of the two- and four-chamber long-axis views (typical parameters: FOV = 440 × 260 mm, matrix = 224 × 90, flip angle = 30°, GRAPPA = 3, TE = 1.0 ms, TR = 2.12 ms, 38 ms per image, 60 images/slice, 8 mm slice thickness, 2 mm gap). Exercise was performed with an MRI-compatible stepping device that enables real-time cardiac imaging during in-magnet exercise (Cardiostep, Ergospect; Innsbruck, Austria). After resting images were acquired, exercise was initiated at 20 watts, with an increase of 5 watts every 20 s with a 40 steps/minute frequency^13^. A study team member provided verbal encouragement from inside the scanner room and collected a rating of perceived exertion (Borg 0–10 scale) at the end of each minute of exercise and after the termination of the exercise test. Peak HR was acquired by finger pulse oximetry. Exercise tests ended when the participant reached volitional exhaustion or could no longer maintain the stepping frequency, followed immediately by acquisition of peak exercise images. A multi-slice, multi-echo acquisition of the right thigh was used to reconstruct water and fat separated images using the modified Dixon approach^14^. Image parameters include 50 axial slices with 4 mm slice thickness (no gap), and 1.0–1.3 mm in-plane resolution.

### Image analysis

T_1_ maps were reconstructed on the scanner and the reported values are the average of all segments from the middle 2 mm layer of the myocardium. We used in-house software (MATLAB, MathWorks, Natick, USA) to measure LV end-diastolic and end-systolic volumes (EDV, ESV) using the bi-plane method of disks^15^ with tracings completed on a single two-chamber and a single four-chamber long-axis image. This method has been previously described in detail and shown to be reliable and reproducible for exercise cardiac function assessment^13^. A complete cardiac cycle was selected for each slice at end-expiration, with manual tracing of the LV endocardium and epicardium on end-diastolic and end-systolic images. SV was measured as the difference between EDV and ESV. Ejection fraction was calculated as SV/EDV. All volumes were normalized to body surface area (BSA)^16^. GLS was calculated as the fractional change in length of the endocardial contour from end-diastole to end-systole relative to end-diastolic length, averaged across the two- and four-chamber long-axis images. For all parameters, reserve was calculated as the difference between peak exercise and resting values.

**Figure 1:**
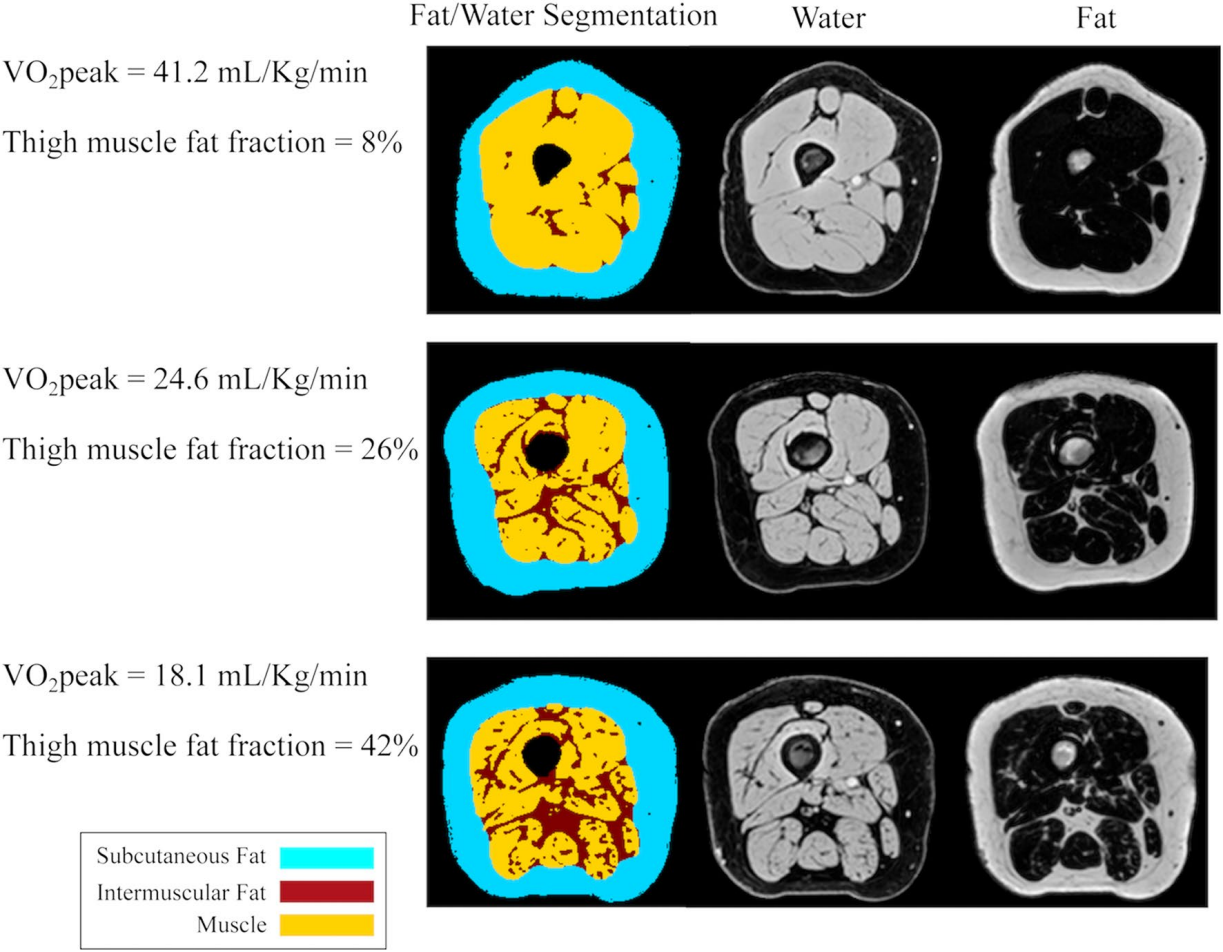
Representative participants with low, medium, and high thigh fat fraction with their respective water and fat suppressed images used to delineate the fat and muscle components. Axial right thigh images were acquired by magnetic resonance imaging and 40 cm of volume were analyzed starting 110 mm from the distal point of the femur to quantify absolute volumes of muscle and intermuscular fat (IMF). The thigh fat fraction was then calculated as: IMF/(IMF + muscle)*100%. Thigh fat fraction was inversely associated with VO_2_peak in both breast cancer survivors and matched controls.

For the thigh images, we used custom semi-automated MATLAB software and the disk summation method for 10 consecutive slices starting 110 mm from the distal femur to measure absolute volumes of muscle and intermuscular fat (IMF), with the femur removed. We calculated the muscle fat fraction as: IMF/(IMF + muscle)*100% (Fig. 1).

### Cardiopulmonary exercise test

The BCS and matched-CON participants performed a graded, to-maximal cardiopulmonary exercise test (CPET) on a cycle ergometer with continuous gas analysis (Encore229 Vmax; SensorMedics, Yorba Linda, USA). The CPET was performed within one week of the MRI scan, or on the same day, ≥ 1-h after the scan. Based on the influence of supine posture in the MRI test^17^, we also assessed upright HR, SV, and CO at rest and during exercise by transthoracic electrical bioimpedance meter (PhysioFlow PF-03, Manatec Biomedical, France)^18^. A finger pulse oximeter was used to collect arterial oxygen saturation (S_a_O_2_) and blood pressure was assessed every two minutes. We allowed ≥ 2 min of rest to ensure stabilization of resting values, then initiated exercise at 20 watts, with 5 watt increments every 20 s (same protocol as MRI). We averaged the impedance data over 30-s epochs from the end of the resting period and the exercise test, as well as the time corresponding to 50 and 75% of VO_2_peak. Peak exercise avO_2_diff was calculated as VO_2_peak divided by peak impedance-derived CO, while systemic vascular resistance (SVR) was calculated as mean arterial pressure divided by impedance-derived CO multiplied by 80.

### Descriptive data

We measured height and weight by electronic scale and stadiometer to calculate BMI, while demographics and cardiovascular risk factors were self-reported. We determined current sedentary status (no moderate or vigorous activity) from the Godin Leisure Time Exercise Questionnaire responses^19^. Study staff extracted diagnosis and treatment information for BCS from medical records. Hemoglobin was measured from a venipuncture taken prior to exercise.

### Statistical analyses

We compared the prevalence of cardiovascular risk factors between groups using Fisher’s exact tests. For comparison between the three groups, we used one-way analysis of variance if the Levene’s and Shapiro–Wilk tests confirmed equal variance and normal distribution, and the Kruskal–Wallis H test otherwise. Independent t-tests or the Mann–Whitney U-test were used for between-group comparisons.

We used linear regression to determine the independent predictive value of the peak exercise cardiac parameters, avO_2_diff, hemoglobin, myocardial T_1_ time, and thigh muscle fat fraction for relative VO_2_peak (mL/kg/min) among the BCS and matched-CON groups combined. An interaction effect between group and each independent variable was included to determine whether cancer status influenced these relationships. For significant interactions, we report the linear relationship separately for the two groups, otherwise the interaction and group effects are removed from the model, leaving a univariate model. When testing cardiac volumes as independent predictors of VO_2_peak, we normalized them to body weight (kilograms) instead of BSA to standardize the units.

To determine whether functional metrics of the myocardium (peak exercise and reserve LV volumes and function) and skeletal muscle (peak avO_2_diff) mediated the linear relationship between myocardial T_1_ time or thigh fat fraction, respectively and relative VO_2_peak, we performed Baron and Kenny’s steps for mediation analysis^20^. In brief, a partial mediator is a variable that: (1) is predicted by the tissue characteristic in linear regression; (2) when adjusted by the tissue characteristic, is a significant linear predictor of VO_2_peak; and (3) reduces the size of the effect of the tissue characteristics on VO_2_peak to nonzero. Partial mediation implies that part of the effect of the tissue characteristic on VO_2_peak is accounted for by the effect of the tissue characteristic on that mediator variable.

Statistical analyses were performed using Python 3.7.0 (Available at http://www.python.org), two-sided p-values of 0.05 to denote statistical significance without adjustments for multiple comparisons.

### Ethics approval

This study was approved by the University of Alberta Research Ethics Board (Pro00040073). Informed consent was obtained from all individual participants included in the study. The procedures used in this study adhere to the tenets of the Declaration of Helsinki.

## Results

### Participants

The BCS and matched-CON groups were similar in demographics, anthropometrics, and cardiovascular risk factors, and by design, the young-CON group had no cardiovascular risk factors, lower BMI and age (Table 1). All participants completed all planned study measures.

**Table 1:** Participant descriptive data.

Variable	Young controls (n = 12)	Matched controls (n = 16)	Breast cancer survivors (n = 16)	p-value
**Demographics and anthropometrics**				
Age (years, mean ± SD)	25 ± 4	56 ± 10*	56 ± 10*	** < 0.001**
Body mass index (kg/m^2^, mean ± SD)	22 ± 2	28 ± 5*	29 ± 4*	** < 0.001**
Body surface area (m^2^, mean ± SD)	1.68 ± 0.10	1.84 ± 0.15*	1.84 ± 0.18*	** < 0.01**
Caucasian (n (%))	12 (100%)	16 (100%)	15 (94%)	1.00

**Cardiovascular risk factors**	**n (%)**	**n (%)**	**n (%)**	**p-value**^**†**^
Sedentary	0	2 (13%)	4 (25%)	0.654
Hypertension^‡^	0	0	2 (13%)	0.484
Hypercholesterolemia	0	2 (13%)	0	0.484
Former smoker	0	8 (50%)	7 (44%)	1.00
Current smoker	0	0	0	-
Diabetes	0	0	0	-
Overweight (25.0–29.9 kg/m^2^)	0	8 (50%)	5 (31%)	0.473
Obese (30.0 + kg/m^2^)	0	7 (44%)	7 (44%)	1.00

**Breast cancer diagnosis and treatment**			**n (%)**	
**Stage**				
I/II			8 (50%)	
III			7 (44%)	
IV			1 (6%)	
**Receptor status**				
Estrogen positive			12 (75%)	
Progesterone positive			8 (50%)	
Human epidermal growth factor 2 positive			0	
Triple negative			4 (25%)	
**Chemotherapy regimen**				
3 × fluorouracil, epirubicin & cyclophosphamide + 3 × docetaxel			14 (88%)	
6 × fluorouracil, epirubicin & cyclophosphamide			1 (6%)	
5 × fluorouracil, doxorubicin & cyclophosphamide			1 (6%)	
Post-intravenous chemotherapy capecitabine			2 (13%)	
**Radiation therapy**				
Left			11 (69%)	
Right			4 (25%)	
**Hormonal therapy (current)**				
Aromatase inhibitor			5 (31%)	
Tamoxifen			4 (25%)	
Both			2 (13%)	

*Statistically different from young controls at p ≤ 0.01.

^**†**^P-value shown for comparison between matched controls and survivors only as young controls lacked all of these risk factors by default.

^‡^Of the two breast cancer group participants reporting a history of hypertension, only one was currently on medication (a calcium channel blocker) and she reported not taking it during anthracycline therapy or on the day of the assessment.

### Supine (MRI) cardiac function

**Figure 2:**
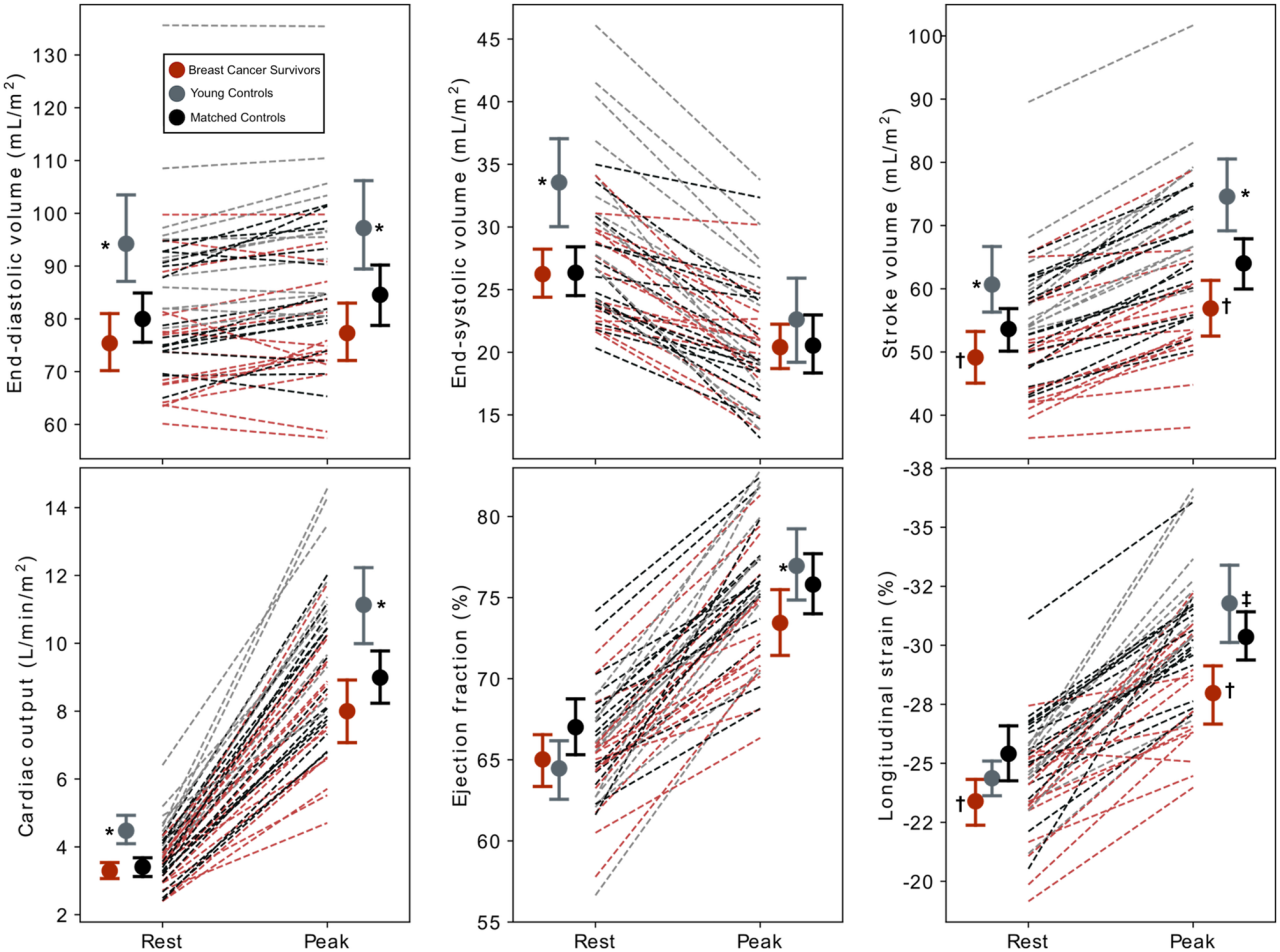
Change and mean cardiac function from rest to peak exercise among anthracycline-treated breast cancer survivors (n = 16), age- and body mass index-matched non-cancer controls (n = 16), and young, healthy controls (n = 12). All data is derived from cardiac magnetic resonance imaging. Volumes are normalized to body surface area. Young controls had larger peak and reserve stroke volume and cardiac output compared to both other groups. Compared to matched controls, breast cancer survivors had lower rest and exercise stroke volume and global longitudinal strain, and cardiac output reserve. *Difference from both breast cancer survivors and matched control groups at p ≤ 0.05; ^**†**^Difference from matched control group at p ≤ 0.05; ^‡^Difference from breast cancer survivor group at p ≤ 0.05;

Individual rest-to-exercise changes in MRI-derived LV volumes and function for all three groups are shown in Fig. 2. Peak power output as well as LV volumes at rest and at peak exercise (except for peak end-systolic volume) were larger in young-CON than both the BCS and matched-CON groups (Table 2). Young-CON also had larger reserve in LV function (ejection fraction, GLS, CO, Table 2). The only difference between BCS and matched-CON, however, was that BCS had lower peak power, lower rest and exercise SV and GLS, and CO reserve (Table 2).

**Table 2:** Cardiac magnetic resonance and cardiopulmonary data.

Variable	Young controls (n = 12) (mean ± SD)	Matched controls (n = 16) (mean ± SD)	Breast cancer survivors (n = 16) (mean ± SD)	P-value
**Resting**				
Heart rate (bpm)	74 Ü9	64 ± 11*	68 ± 10	**0.042**
End-diastolic volume index (mL/m^2^)	94 ± 15	80 ± 10*	75 ± 12*	**0.001**
End-systolic volume index (mL/m^2^)	34 ± 7	26 ± 4*	26 ± 4*	** < 0.001**
Stroke volume index (mL/m^2^)	61 ± 10	54 ± 7*	49 ± 9*^†^	**0.009**
Ejection fraction (%)	64 ± 3	67 ± 4	65 ± 3	0.123
Cardiac output index (L/min/m^2^)	4.5 ± 0.8	3.4 ± 0.6*	3.3 ± 0.5*	** < 0.001**
Global longitudinal strain (%)	− 24.4 ± 1.4	− 25.4 ± 2.4	− 23.4 ± 2.2^†^	**0.032**
**Peak supine MRI exercise**				
Supine stepper power output (watts)	255 + 25	151 ± 29*	122 ± 30*^†^	** < 0.001**
Heart rate (bpm)	155 ± 22	140 ± 17	140 ± 17	0.067
End-diastolic volume index (mL/m^2^)	97 ± 15	85 ± 11*	77 ± 12*	** < 0.001**
End-systolic volume index (mL/m^2^)	23 ± 6	21 ± 5	20 ± 4	0.448
Stroke volume index (mL/m^2^)	75 ± 11	64 ± 9*	57 ± 10*^†^	** < 0.001**
Ejection fraction (%)	77 ± 4	76 ± 4	73 ± 4	0.071
Cardiac output index (L/min/m^2^)	11.1 ± 2.2	9.0 ± 1.7*	8.0 ± 2.0*	** < 0.001**
Global longitudinal strain (%)	− 31.8 ± 3.0	− 30.4 ± 2.2	− 28.0 ± 2.5*^†^	**0.001**
**Reserve (peak–rest)**				
Heart rate (bpm)	81 ± 19	76 ± 12	72 ± 20	0.394
End-diastolic volume index (mL/m^2^)	3 ± 3	5 ± 5	2 ± 6	0.312
End-systolic volume index (mL/m^2^)	− 11 ± 3	− 6 ± 3*	− 6 ± 4*	** < 0.001**
Stroke volume index (mL/m^2^)	14 ± 4	10 ± 4*	8 ± 4*	**0.001**
Ejection fraction (%)	12 ± 3	9 ± 3*	8 ± 3*	**0.004**
Cardiac output index (L/min/m^2^)	6.7 ± 2.0	5.6 ± 1.3*	4.7 ± 1.6*^†^	**0.012**
Global longitudinal strain (%)	− 7.4 ± 2.1	− 5.0 ± 2.0*	− 4.6 ± 2.5*	**0.003**
**Cardiopulmonary cycle exercise test data**				
Resting arterial oxygen saturation (%)	–	98 ± 2	97 ± 2	0.357
Peak arterial oxygen saturation (%)	–	94 ± 2	96 ± 2	0.080
Peak heart rate (bpm)	–	161 ± 14	166 ± 12	0.110
Peak power output (watts)	–	165 ± 30	132 ± 31	**0.004**
Peak oxygen consumption (L/min)	–	2.13 ± 0.41	1.69 ± 0.37	**0.003**
Peak oxygen consumption (mL/kg/min)	–	29.5 ± 7.7	23.1 ± 7.5	**0.011**
Peak respiratory exchange ratio	–	1.27 ± 0.08	1.29 ± 0.09	0.600
Arteriovenous oxygen difference (mL/100 mL, impedance-derived)	–	16.4 ± 3.6	15.2 ± 3.9	0.054

*Statistically different from young controls at p ≤ 0.05; ^†^ Statistically different from matched controls at p ≤ 0.05;– denotes data not collected in this group.

At peak exercise in the CMR, all participants reported a peak rating of perceived exertion of ≥ 9 on the Borg 0–10 scale. The peak power output achieved in the supine CMR stepper exercise test corresponded to 93 ± 11% of that achieved on the upright cycle ergometer test.

### Upright (impedance cardiography) cardiac function

**Figure 3:**
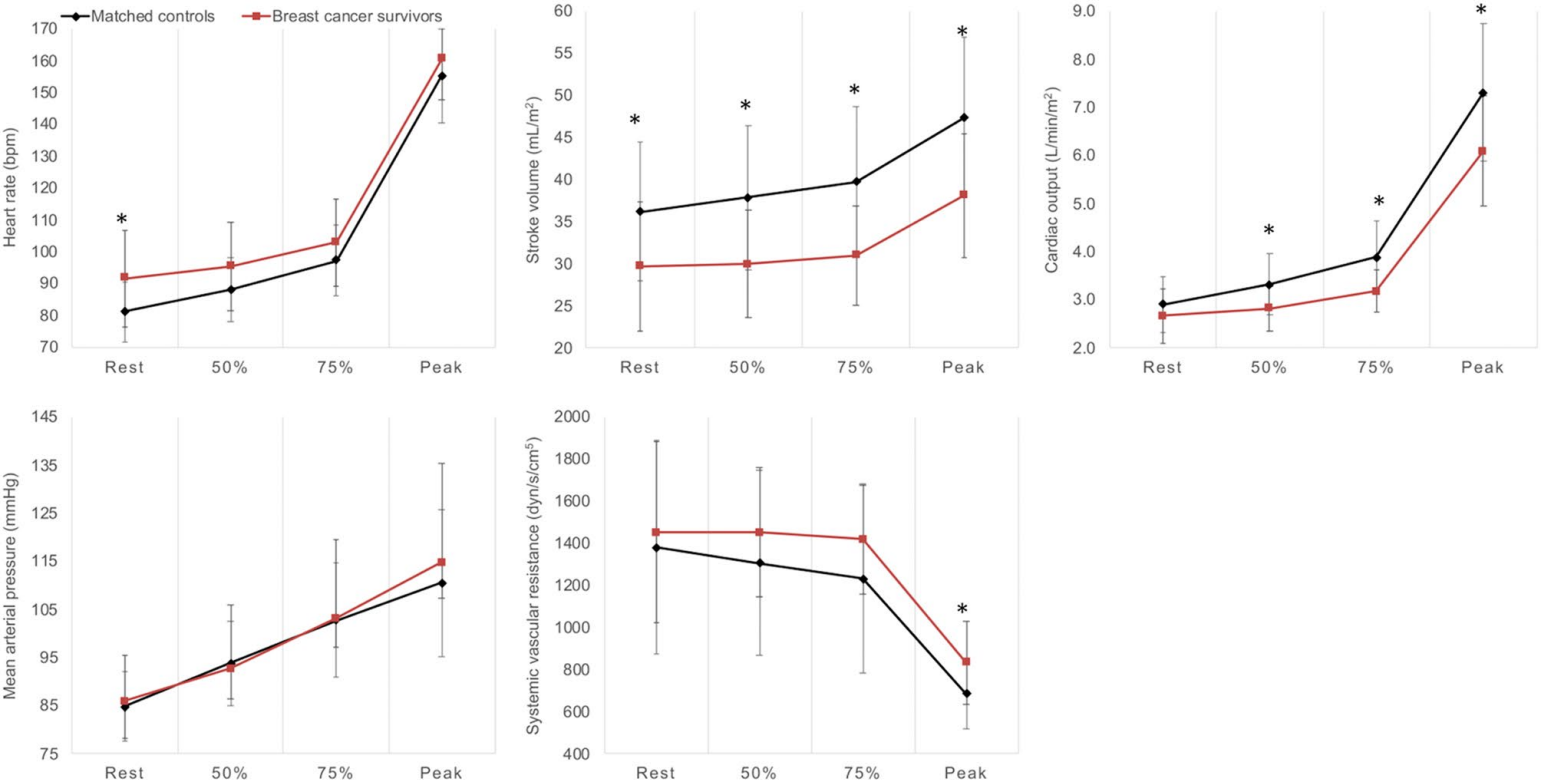
Bioelectrical impedance cardiography data for cycle ergometer graded exercise test in breast cancer survivors (n = 16), age- and body mass index-matched non-cancer controls (n = 16). Stroke volume and cardiac output are normalized to body surface area. *Difference between groups at p ≤ 0.05.

Upright cycle ergometer responses of HR, SV, CO, and SVR assessed by impedance cardiography were compared between BCS and matched-CON at rest, 50%, 75%, and 100% of VO_2_peak. There were similar differences between BCS and matched-CON for upright SV and CO to supine data (Fig. 3). At peak exercise, calculated avO_2_diff was lower and SVR was higher in BCS versus matched-CON (Table 2, Fig. 3, respectively).

### Hemoglobin and VO_2_ peak

Hemoglobin did not differ between BCS and matched-CON (133 ± 11 vs 134 + 8, p = 0.686). All participants exceeded a respiratory exchange ratio of 1.10 on the maximal cardiopulmonary exercise test and reached volitional exhaustion. Absolute and relative VO_2_peak were lower in BCS versus matched-CON (Table 2).

### Determinants of VO_2_ peak

**Figure 4:**
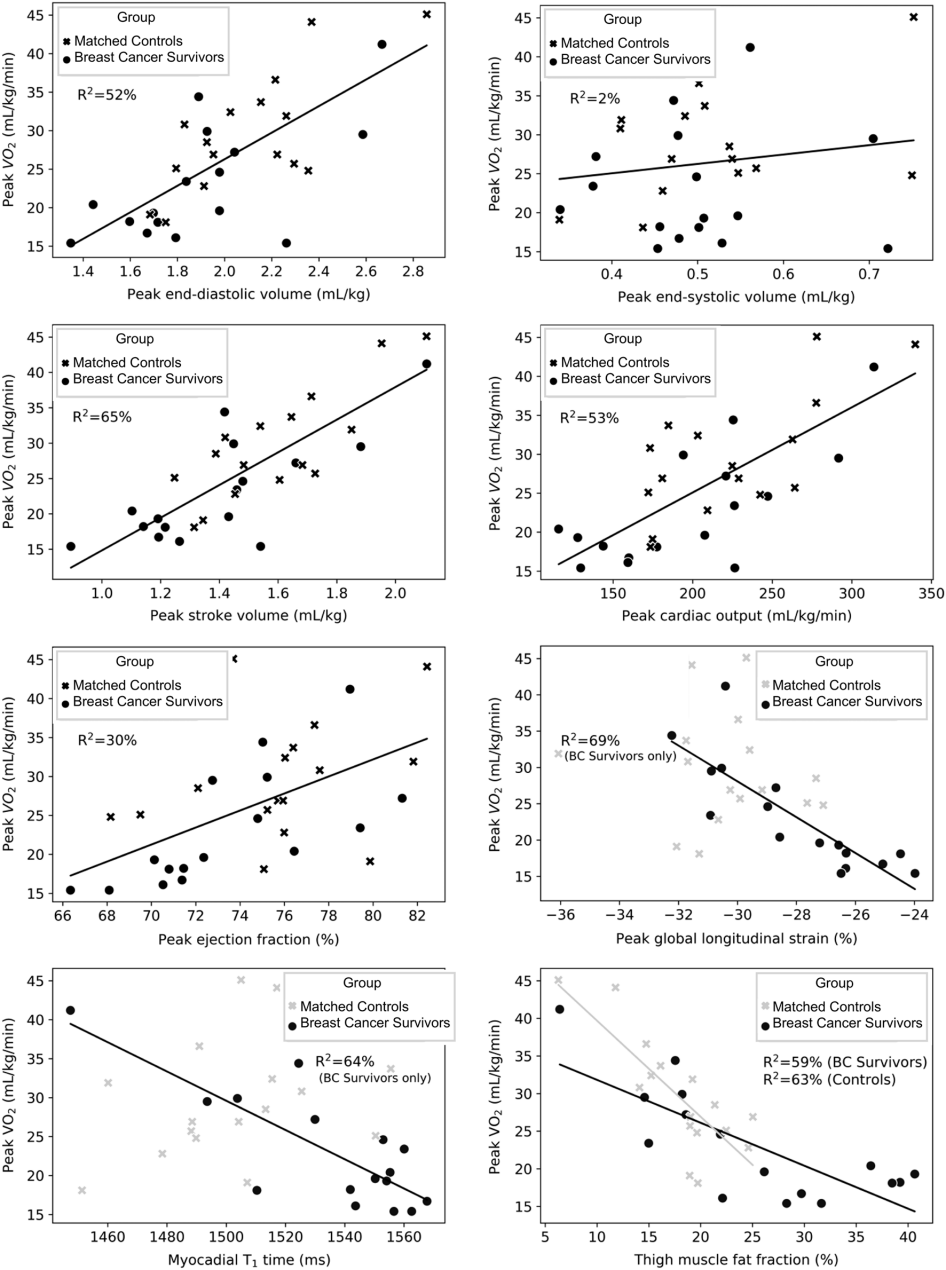
Predictors of peak volume of oxygen consumption (VO_2_) among anthracycline-treated breast cancer survivors and age- and body mass index-matched non-cancer controls. Trend line colors match included data. Both VO_2_ and volumes are normalized to body weight (kg) to standardize units.

Peak exercise GLS (more negative is better function) and myocardial T_1_ time (lower is less fibrosis) were inversely correlated with VO_2_peak in BCS (ß = − 2.5, 95% CI − 3.4, − 1.5, p < 0.001, R^2^ = 69%, ß = − 0.19, 95% CI − 0.27, − 0.11, p < 0.001, R^2^ = 64%, respectively; Fig. 4) and were not related in matched-CON. Thigh muscle fat fraction had a statistically different relationship with VO_2_peak in the two groups that was similar in direction and strength (BCS: ß = − 0.6, 95% CI − 0.8, − 0.3, p < 0.001, R^2^ = 59; matched-CON: ß = − 1.3, 95% CI − 1.8, − 0.7, p < 0.001, R^2^ = 63%, both p < 0.001), but with a steeper slope in the matched-CON group (Fig. 4). Peak exercise EDV, SV, CO, EF, and avO_2_diff were significant predictors of VO_2_peak with no influence of group (Table 3).

**Table 3:** Predictors of relative VO_2_peak (mL/kg/min) for breast cancer and matched controls combined (n = 32) with adjustment for group.

Variables differing by group:	P-value: group interaction	ß (95% CI) for breast cancer	R^2^ (%)
Peak global longitudinal strain (%)	**0.048**	− 2.5 (− 3.8, − 1.1)	45
Myocardial T_1_ time (ms)	**0.002**	− 0.19 (− 0.29, − 0.08)	46
Thigh muscle fat fraction (%)	**0.022**	− 0.57 (− 0.83, − 0.32)	68

Variables without influence of group:	P-value: main effect	ß (95% CI) for both groups	R^2^ (%)
Peak end-diastolic volume (mL/kg)	** < 0.001**	17.2 (11.1, 23,4)	52
Peak end-systolic volume (mL/kg)	0.394	12.0 (− 16.4, 40.5)	2
Peak stroke volume (mL/kg)	** < 0.001**	23.1 (16.8, 29.4)	65
Peak cardiac output (mL/kg/min)	** < 0.001**	0.11 (0.07, 0.15)	53
Peak supine heart rate (beats per minute)	0.103	0.14 (− 0.03, 0.32)	9
Peak upright heart rate (beats per minute)	**0.050**	0.22 (0, 0.44)	12
Peak ejection fraction (%)	**0.001**	1.1 (0.5, 1.7)	30
Hemoglobin (g/dL)	0.248	1.8 (− 1.3, 5.0)	4
Peak arteriovenous oxygen difference (mL/100 mL, impedance-derived)	** < 0.001**	1.3 (0.7, 2.0)	37

Bolding indicates significance.

*ß* unstandardized beta coefficient, *CI* confidence interval, *R*^*2*^ coefficient of variation.

### Is the effect of cardiac or skeletal muscle tissue characteristics on VO_2_peak mediated by function of these tissues?

We used mediation analysis to test the hypothesis that the relationships between myocardial T_1_ time and thigh muscle fat fraction with VO_2_peak are mediated by function of these tissues in BCS. Myocardial T_1_ time was a significant predictor of peak exercise EDV, SV, and CO, as well as reserve in ESV and CO. Of these variables meeting the first criterion in mediation analysis, only reserve in CO and ESV also partially reduced the effect size of myocardial T_1_ on VO_2_peak (second criterion) and remained significant predictors of VO_2_peak (final criterion). In other words, part of the effect of myocardial T_1_ time on VO_2_peak is accounted for by the effect of myocardial T_1_ time on CO reserve and ESV reserve. Likewise, avO_2_diff met the criteria as a partial mediator of the effect of thigh muscle fat fraction on VO_2_peak.

## Discussion

This comprehensive MRI study produced several novel findings regarding exercise cardiac function and peripheral predictors and mediators of VO_2_peak as well as potential underlying mechanisms among anthracycline-treated BCS.

Firstly, we substantially extend previous findings on exercise cardiac function in BCS^2,21^, by reporting that submaximal and peak exercise SV are blunted in both the supine (by MRI) and upright (by impedance cardiography) body positions among anthracycline-treated BCS compared to non-cancer controls matched for cardiovascular risk factors. Second, our comparison of young- and matched-CON demonstrates that traditional cardiovascular risk factors (age, overweight/obesity, lifestyle behaviors) without cardiotoxic treatment result in reduced exercise reserve of LV volumes and function. The only additional cardiac impairment in the anthracycline-treated BCS group, who were otherwise closely balanced with matched-CON on all cardiovascular risk factors, was blunted SV (a direct determinant of VO_2_peak) and GLS (a sensitive predictor of cardiotoxicity when measured at rest)^7^. Novel findings related to peak exercise GLS were that it correlated with VO_2_peak in the BCS group only, and that GLS reserve was greater in young-CON, but similar between BCS and matched-CON. The longitudinal shortening of the LV is a primary determinant of SV^6^, and all of our GLS findings paralleled those for SV. Together, these novel exercise GLS findings suggest that impaired longitudinal shortening may be an important myocardial mechanism for smaller exercise SV and resultant VO_2_peak among both anthracycline-treated and cardiovascular risk female populations.

In this study, similar to previous reports^2,10,22^, VO_2_peak was ~ 20% lower in BCS compared to matched-CON. Our novel peak exercise cardiac MRI and muscle tissue characterization data indicate that central and peripheral factors explain similar amounts of variation in VO_2_peak in anthracycline-treated BCS. Further, by mediation analyses, we explored the mechanism or process by which cardiac and skeletal muscle tissue characteristics may influence VO_2_peak through associations with function. We determined that the association of native myocardial T_1_ time, where longer times are associated with a greater degree of biopsy-quantified fibrosis^23^, on VO_2_peak is partially explained by the association of T_1_ time on the reserve of CO or more specifically, ESV. As EDV (and likely preload) did not appreciably change, in line with previous supine MRI results in other populations^24^, the reduction in ESV is the primary driver of the exercise-induced increase in SV. As a reduction in ESV with acute exercise results from increased inotropy and/or reduced afterload, this finding suggests that within BCS, myocardial fibrosis and potentially the associated loss in healthy cardiac muscle fibers impairs one or both of these, which in turn leads to lower VO_2_peak. Indeed, surrogate measures (GLS and SVR) were impaired, on average, in the BCS group.

We also determined that the negative association of fatty infiltration of the thigh with VO_2_peak is partially explained by a negative association with avO_2_diff. This finding is in line with our previous finding for non-cardiac limited, single-leg, plantar flexion exercise, where a greater ratio between IMF and muscle in the lower leg was associated with multiple aspects of diffusive oxygen transport and/or utilization including local oxygen consumption and extraction, and blood flow^10^. Elevated levels of IMF and visceral fat at the time of breast cancer diagnosis have been associated with elevated future incidence of cardiovascular disease^25^. Post-anthracyclines, BCS also have greater visceral fat compared to controls, despite similar amounts of subcutaneous fat^10^. Given that both IMF and visceral fat are associated with insulin resistance^26,27^, this suggests that BCS exhibit a detrimental cardiometabolic phenotype. It should be noted that the relationship between thigh muscle fat fraction and VO_2_peak also existed in matched-CON, who were primarily older women with overweight/obesity. This suggests that fatty infiltration of the muscles is an underappreciated determinant of cardiovascular risk in women in general.

Future studies are required to quantify the longitudinal impact of breast cancer treatment on peak SV and IMF and to evaluate interventions to mitigate injury during treatment or improve these metrics post-treatment. Given that myocardial fibrosis is thought to be irreversible, prophylactic strategies during cardiotoxic treatment may be needed to prevent myocardial injury and preserve central determinants of VO_2_peak. In contrast, IMF is amenable to interventions such as caloric restriction and exercise training in other populations^26^, so this plasticity may enable intervention timing either during or after cancer treatment as strategies to improve VO_2_peak.

A limitation of this study is that the cross-sectional design does not allow determination of causal influences. Our sample size is small, however, our comprehensive assessment using highly reproducible MRI techniques allows the detection of meaningful differences with a sample size as small as 15 per group^28^. Further we have recently reported the excellent reliability and reproducibility of our exercise CMR technique^15^. Ideally the MRI measures of cardiac function and VO_2_peak would have been performed in the same body position, but our upright cardiac impedance-derived measures demonstrated the same trend. Finally, generalizability is limited to primarily Caucasian and middle-aged BCS.

In conclusion, among BCS who are post-anthracycline treatment, peak exercise SV and CO and skeletal muscle oxygen extraction are blunted compared to matched-CON and are both important determinants of VO_2_peak. Mediation analysis showed that greater myocardial fibrosis is associated with impaired exercise reserve ESV and CO which is related to reduced VO_2_peak. Greater thigh muscle fatty infiltration is associated with impaired oxygen extraction which is also related to reduced VO_2_peak. Fatty infiltration of the thigh muscle may be an underappreciated determinant of cardiovascular risk in women with and without breast cancer. Given that myocardial fibrosis is considered irreversible, but fatty infiltration of muscle is plastic, strategies to improve exercise capacity in anthracycline-treated BCS could include interventions to prevent myocardial injury during treatment and/or that improve fatty infiltration either during or after treatment.

## Data Availability

The datasets generated during and/or analysed during the current study are available from the corresponding author on reasonable request.
